# Investigation of the Copper Requirements of the Metallophyte Liverworts *Cephaloziella nicholsonii* Douin and *C. massalongoi* (Spruce) Müll.Frib

**DOI:** 10.3390/plants12122265

**Published:** 2023-06-09

**Authors:** Christina Campbell, Daniel L. Kelly, Noeleen Smyth, Neil Lockhart, David T. Holyoak, David Long

**Affiliations:** 1 National Botanic Gardens of Ireland, Glasnevin, D09 VY63 Dublin 9, Ireland; 2 Department of Botany, Trinity College, University of Dublin, D02 PN40 Dublin 2, Ireland; daniel.kelly@tcd.ie; 3 School of Agriculture and Food Science, University College Dublin, Belfield, D04 V1W8 Dublin 4, Ireland; noeleen.smyth@ucd.ie; 4 National Parks and Wildlife Service, 90 King Street North, D07 N7CV Dublin 7, Ireland; neil.lockhart@npws.gov.ie; 5 Independent Researcher; holyoak9187@gmail.com; 6 Royal Botanic Garden, Edinburgh EH3 5LR, UK; d.long@rbge.ac.uk

**Keywords:** liverwort, metallophyte, in vitro cultivation, heavy metals, conservation

## Abstract

Former mine sites can provide habitat for many rare specialised bryophyte species that have adapted to metal-rich soil conditions that are toxic to most other plant species. Some of the bryophyte species found in this habitat are facultative metallophytes, and others are regarded as strict metallophytes, the so-called ‘copper mosses’. It is a general assumption in the literature that *Cephaloziella nicholsonii* and *C. massalongoi*, both categorised as Endangered in the IUCN Red List for Europe, are also strict metallophytes and obligate copper bryophytes. This in vitro experiment investigated the growth and gemma production of these two species from different sites in Ireland and Britain on treatment plates of 0 ppm, 3 ppm, 6 ppm, 12 ppm, 24 ppm, 48 ppm and 96 ppm copper. Results show that elevated copper is not an obligate requirement for optimum growth. Differences in response to the copper treatment levels among populations evident within both species could possibly be due to ecotypic variation. A case is also made for the taxonomic revision of the *Cephaloziella* genus. Implications for the species’ conservation are discussed.

## 1. Introduction

Old mine workings and mine-spoil heaps containing high levels of heavy metals, such as copper, lead and zinc, provide a habitat for many species of specialised bryophytes [1,2,3]. The vegetation of mine-waste sites is ascribable to the habitat ‘Calaminarian Grassland of the *Violetalia calaminariae*’, a habitat listed in Annex I of the EU Habitats Directive [4]. The equivalent National Vegetation Classification (NVC) category in Britain is the Festuca-Minuartia community (OV37) [5]. In Ireland, this habitat has been linked with the phytosociological association Minuartio-Thlaspietum alpestris Koch 1932 in the class *Violetea calaminariae* [6]. In some parts of Europe, this habitat can occur naturally on outcrops of metal ores, but in Ireland and Britain sites with this habitat are mainly confined to former mine workings and spoil heaps [7]. The soils are generally low in nutrients, have a poorly developed structure and are often drought-prone [8].

Available copper in uncontaminated soils is generally <100 ppm [9,10] and soil is considered heavily contaminated with available copper at levels >500 ppm [10]. The levels of copper at many former mine sites in Ireland and Britain exceed the latter value [3,11]. Plant toxicity thresholds for copper in soil are generally in the range of 20–850 ppm [12] and the levels of metals present in mine spoil are toxic to many species of angiosperm [13], apart from some facultative metallophytes, such as *Agrostis capillaris*, *Armeria maritima* and *Silene uniflora* [14]. However, these former mine sites support a number of rare and specialised metallophyte bryophytes characteristic of the Calaminarian Grassland habitat in Ireland and Britain, such as the liverwort species *Cephaloziella stellulifera*, which can dominate large (several metres squared) areas [15,16,17]. Unlike tracheophytes, bryophytes lack a root system and can absorb substances across their entire body surface. In general, they are more reliant on water from the atmosphere than from their substratum. Bryophytes accumulate heavy metals by several mechanisms, including adsorption on the cell surface; tolerances vary among species, as well as among metal types [18].

Some of the bryophytes found in the habitat are facultative metallophytes, such as *Cephaloziella integerrima*, *C. stellulifera*, *Ceratodon purpureus*, *Gymnocolea inflata*, *Pohlia andalusica*, *Scapania compacta*, *Solenostoma gracillimum* and *Weissia controversa* var. densifolia [14,16]. Others are regarded as strict metallophytes, many of which are of high conservation value: the obligate lead moss, *Ditrichum plumbicola* [14,19] and the so-called ‘copper mosses’, such as *Mielichhoferia elongata* [20], *M. mielichhoferi* [21], *Ditrichum cornubicum* [22,23,24,25] and *Scopelophila cataractae* [26,27]. The term ‘copper moss’ has also been extended to include liverworts, including the two species investigated here, *Cephaloziella nicholsonii* Douin and *C. massalongoi* (Spruce) Müll.Frib.

*C. massalongoi* has been assigned to the Suboceanic Boreo-temperate geographical element in Europe [28,29]; it is recorded from Austria, Bulgaria, Corsica, Finland, France, Germany, Iceland, Ireland, Italy, Norway, Portugal, Romania, Slovakia, Spain, Sweden, Switzerland and the UK [30,31]. It has also been documented in Japan, Nepal, Canada and the United States of America [32,33,34,35]. In Europe, *C*. *nicholsonii* is classed as a Suboceanic Temperate species [28,29] and, outside Ireland and Britain, only an old (1936) record from Germany exists [36]. *C. massalongoi* and *C. nicholsonii* have been recorded from six and seven localities in Ireland respectively [14,17,37,38,39], and both species are known from several localities in the UK in the former copper-mining regions of south-west England and north-west Wales [28,40]. *C. massalongoi* has been reported from naturally metal-rich sites in Europe, e.g., in the Pyrenees [41,42], but in Ireland and Britain, it is only known from sites in the vicinity of disused copper mines in association with metalliferous rocks, soils and mine waste. *C. nicholsonii* occurs in similar habitats to *C. massalongoi*, but is considered somewhat more desiccation tolerant [40]. Both species are classified as Endangered on the IUCN European Bryophyte Red List [43] and Vulnerable in Ireland [14], while in the UK *C. nicholsonii* is classified as Endangered and *C. massalongoi* as Vulnerable [44]. Both species are threatened at their sites in Ireland and Britain by reclamation and coverage with top-soil, dumping and eventual copper leaching leading to recolonisation by angiosperms and shading out [14,40]. *C. nicholsonii* has a chromosome number of 36, whereas *C. massalongoi* has 18 chromosomes, and it is thought that *C. nicholsonii* may have evolved from *C. massalongoi* through the doubling of the chromosome number [45,46].

It is a general assumption that *C. nicholsonii* and *C. massalongoi* are strict metallophytes and obligate copper bryophytes [14,16,37]. Both species have consistently been found on soils with levels of copper that are toxic to normal plant growth [3,14,47]. However, this assumption has not been investigated by experimental means. There is anecdotal evidence that *C. massalongoi* may not be a strict metallophyte, as records exist for it from substrates with low copper concentrations [25,48].

While a number of studies have focussed on the effects and responses to elevated heavy metal concentrations on mosses, few have investigated the effects on liverwort species [49]. In the current study, we use in vitro cultivation, an ex-situ technique that involves growing species in tissue culture under sterile conditions [50]. This technique has many advantages: development from initial spore germination to sporophyte production can be studied closely and it allows experimental investigation under controlled conditions of a wide variety of ecological issues, including heavy metal tolerance [51,52,53]. In this experiment, samples of *Cephaloziella nicholsonii* and *C. massalongoi* from three populations each from Ireland and Britain were cultured on treatment plates of 0 ppm, 3 ppm, 6 ppm, 12 ppm, 24 ppm, 48 ppm and 96 ppm copper. The aims of the experiment were to investigate the levels of copper required by both species and to test the hypothesis that they do not merely tolerate higher levels of copper than other species—and thus succeed due to lack of competition—but require raised levels of it for growth. The findings will inform the optimum copper levels for species growth and production of gemmae to maintain these rare species in ex-situ culture and also provide data on the species’ copper requirements for their in situ conservation.

Taxonomy and nomenclature follow Blockeel et al. [54].

## 2. Results

### 2.1. Soil Analysis

Results of soil analyses and additional site details are presented from the Irish sites for *Cephaloziella nicholsonii* from Caim, Co. Wexford and Allihies, Co. Cork and for *C. massalongoi* from Ross Island, Co. Kerry, Glendasan, Co. Wicklow, Bunmahon, Co. Waterford and Allihies, Co. Cork, and from British sites for *C. nicholsonii* at South Caradon Mine, Cornwall and for *C. massalongoi* at Gunnislake Clitters, Cornwall (Table 1).

### 2.2. Growth through Course of Experiment

**Figure 1:**
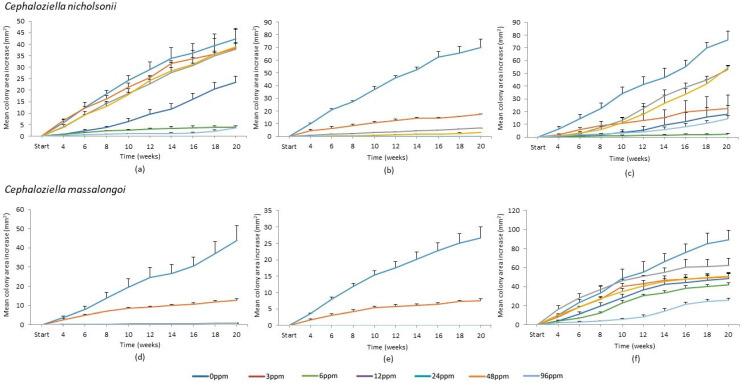
Mean colony area (mm^2^) minus the proportion of initial colony area per measurement, with standard error bars, shown over time for *Cephaloziella nicholsonii* from (**a**) Allihies Mountain Mine, Co. Cork, (**b**) Caim, Co. Wexford and (**c**) South Caradon Mine, Cornwall and for *C. massalongoi* from (**d**) Ross Island, Co. Kerry, (**e**) Glendasan, Co. Wicklow and (**f**) Gunnislake Clitters, Cornwall.

When inspected one week after the commencement of the experiment, plates of *C. massalongoi* from Gunnislake Clitters and some plates of *C. nicholsonii* from Caim were somewhat infected with white fungal hyphae. Measurement and analysis on these plates continued, but it must be borne in mind that fungal growth may have negatively affected the growth of the target species. No growth had occurred of the separate additional populations of *C. massalongoi* from Allihies and Bunmahon at any time during the course of the experiment. Cells turned hyaline within a month on these plates. These plates were excluded from further measurement and analysis. By week 20, an advancement to a plateau in growth was seen in some replicates that had been growing steadily, and a few stems of replicates that had been growing well began to wither and turn white, and so the experiment was terminated. For graphical display, all fortnightly measurements minus the proportion of initial area, are shown for each population in Figure 1.

### 2.3. Cephaloziella nicholsonii

**Figure 2:**
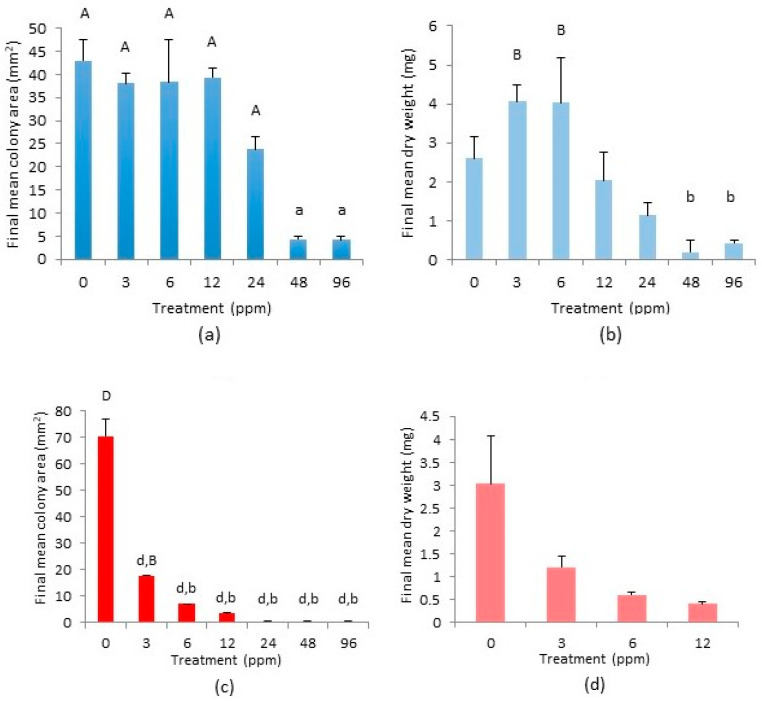

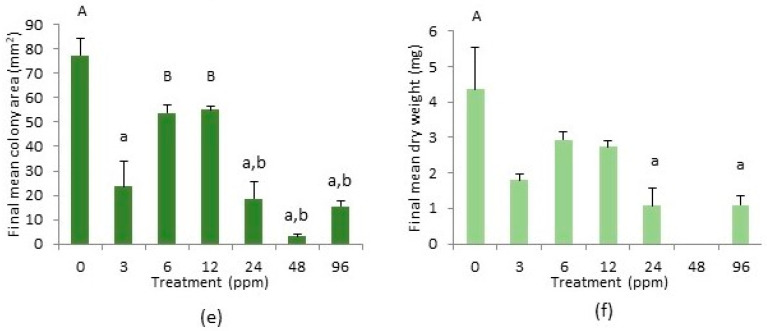
Final mean colony area (mm^2^) and final mean dry weight (mg) per copper treatment, with standard error bars, at week 20 for *Cephaloziella nicholsonii* from (**a**,**b**) Allihies, (**c**,**d**) Caim (only dry weights of treatments 0–12 ppm treatments are shown as no growth occurred on the 24–96 ppm treatments) and (**e**,**f**) South Caradon (final mean dry weight of the 48 ppm treatment omitted from analysis). Columns marked ‘A’ are significantly greater than all columns marked ‘a’ (*p* < 0.01), columns marked ‘B’ are significantly greater than those marked ‘b’ (*p* < 0.05) and columns marked ‘D’ are significantly greater than those marked ‘d’ (*p* < 0.0001).

Growth, in terms of increase in colony area (mm^2^), occurred in all cultures of *C. nicholsonii* from Allihies and was still increasing at the end of the experiment. Growth on the 48 ppm treatment increased somewhat over time but began to show signs of morbidity, i.e., the stems turned hyaline, around week 12 (Figure 1a). Thereafter, only the tips were green and continued to extend very little in comparison with the 96 ppm replicates. The tips of the 96 ppm shoots remained green throughout and began to grow at an increased rate from week 16 on. Colonies on the 3 ppm treatment plates began increasing in the area more than on other treatment plates, but towards the end of the experiment the colonies became vitrified and expansion in area decreased. Gemmae were observed on the colonies on the 0 ppm plates only. The mean increase in colony areas (mm^2^) of replicates grown on the 48 ppm and 96 ppm treatments were significantly less than those on the other treatments (*p* < 0.01) (Figure 2a). However, only the mean dry weight (mg) of colonies on the 3 ppm and 6 ppm treatments were found to be significantly more than those on the 48 ppm and 96 ppm treatment plates at *p* < 0.05 (Figure 2b).

Replicates of *C. nicholsonii* from Caim on the 0 ppm, 3 ppm, 6 ppm and 12 ppm treatments grew steadily (albeit at different rates) until the termination of the experiment, despite some replicates being infected with a fungus. Replicates on the 24 ppm, 48 ppm and 96 ppm copper treatments failed to grow and appeared to have died by week four (Figure 1b). Some of these plates were also infected with a fungus by week four, but growth did not occur on uninfected plates either. Gemmae were observed on the 0 ppm and 3 ppm treatment plates only. The mean colony area increase (mm^2^) on the 0 ppm treatment was significantly greater than on all other treatment plates (*p* < 0.0001). The mean colony area on the 3 ppm copper treatment was significantly greater than on the 12 ppm, 24 ppm, 48 ppm and 96 ppm treatments (*p* < 0.05) (Figure 2c). There was no significant difference between the mean colony areas on the 6 ppm and 12 ppm treatments and, even though some growth did occur on these plates, the mean colony area was not significantly greater than the initial area on plates of the 24 ppm, 48 ppm and 96 ppm treatments (Figure 2c). Regarding dry weights (mg), Scheffé post-hoc tests did not reveal any significant differences between the treatments (Figure 2d), although the 12 ppm treatment dry weight was almost significantly less than that of the control (*p* = 0.07).

Growth on each treatment from the South Caradon population continued throughout the experiment until its conclusion (Figure 1c), except for the replicates on the 48 ppm treatment which by week 18 had started to show signs of necrosis. Colonies on the 3 ppm treatment again began growing well and continued growing, although from about week 10, they began to show signs of vitrification. Gemmae were observed on all treatment plates apart from the 48 ppm plates. Growth, as measured by final mean colony area (mm^2^), was significantly greater on the 0 ppm treatment than on the 3 ppm, 24 ppm, 48 ppm and 96 ppm treatments (*p* < 0.01) and was also greater on the 6 ppm and 12 ppm treatments relative to the 24 ppm and 96 ppm treatments (*p* < 0.05) and to the 48 ppm treatments (*p* < 0.01) (Figure 2e). Final dry weight (mg) was significantly higher on the 0 ppm treatment than on the 24 ppm and 96 ppm treatments (*p* < 0.05) according to the Scheffé post-hoc test (Figure 2f). The dried colonies of the 48 ppm treatment replicates were too minuscule to register on the analytical balance and so were omitted from the ANOVA.

**Figure 3:**
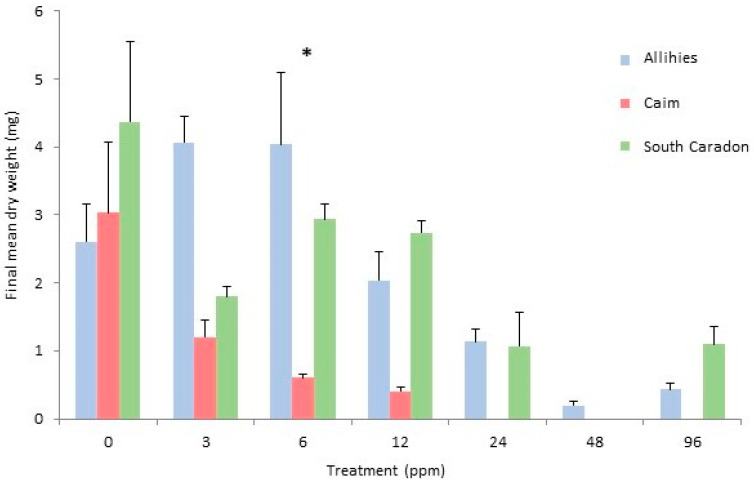
Final mean dry weight (mg) per copper treatment, with standard error bars, at week 20 for *Cephaloziella nicholsonii* from all three populations, Allihies, Caim and South Caradon. A significant difference was found among populations within the treatment marked ‘*’ (*p* < 0.05).

Two-way factorial ANOVA (analysis of variance) showed there was an overall significant difference among the three populations (Table 2) and Scheffé post-hoc tests revealed that the final dry weights of both the Allihies and the South Caradon Mine populations were significantly greater than the Caim population overall, with no significant difference between the two aforementioned populations. There was also an overall treatment effect and Scheffé post-hoc tests showed that the 0 ppm treatment results were significantly greater than those of the 12 ppm, 24 ppm, 48 ppm and 96 ppm treatments, the 3 ppm treatment results were significantly greater than those of the 24 ppm, 48 ppm and 96 ppm treatments, the 6 ppm treatment results were significantly greater than those of the 24 ppm, 48 ppm and 96 ppm treatments, and the 12 ppm treatment result was significantly greater than that of the 48 ppm treatment. Scheffé post-hoc tests also revealed that within the 6 ppm treatment, the mean dry weight of the Allihies population was significantly greater than that of the Caim population (*p* < 0.05) (Figure 3).

### 2.4. Cephaloziella massalongoi

**Figure 4:**
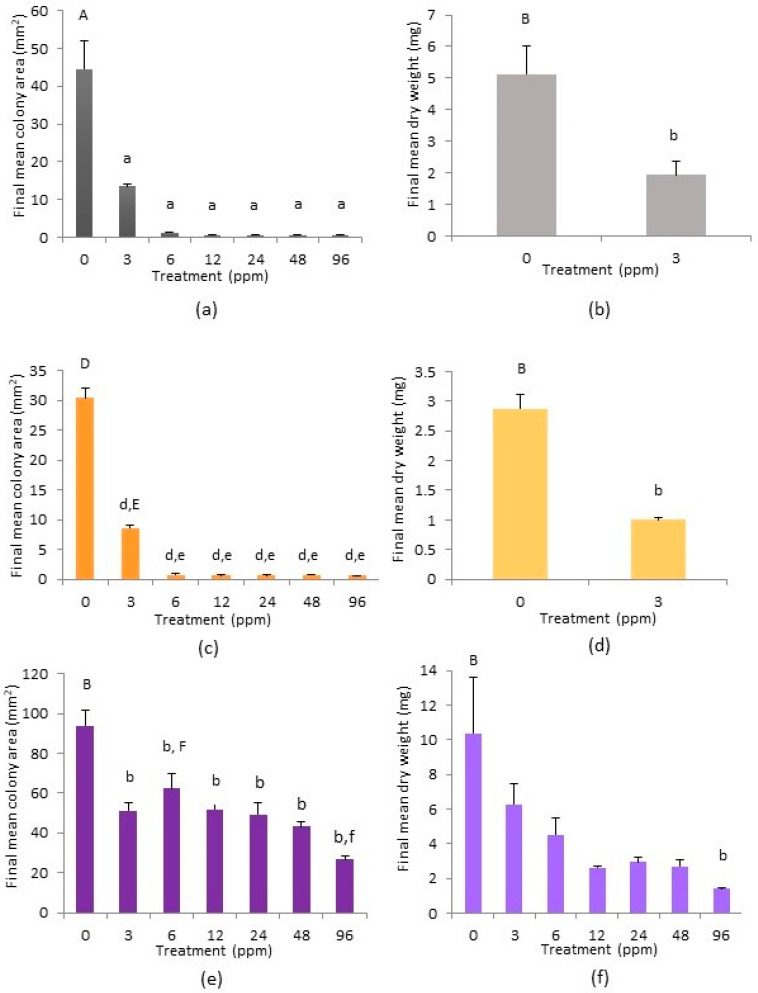
Final mean colony area (mm^2^) and final mean dry weight (mg) per copper treatment, with standard error bars, at week 20 for *Cephaloziella massalongoi* from (**a**,**b**) Glendasan, (**c**,**d**) Ross Island (only dry weights of treatments 0 ppm and 3 ppm treatments are shown as no growth occurred on the 6–96 ppm treatments) and (**e**,**f**) Gunnislake Clitters (dry weights of treatments 0 ppm and 3 ppm treatments are solely shown as no growth occurred on the 6–96 ppm treatments). Columns marked ‘A’ are significantly greater than all columns marked ‘a’ (*p* < 0.01), columns marked ‘B’ are significantly greater than those marked ‘b’ (*p* < 0.05), columns marked ‘D’ are significantly greater than those marked ‘d’ (*p* < 0.001), columns marked ‘E’ are significantly greater than those marked ‘e’ (*p* < 0.001) and columns marked ‘F’ are significantly greater than those marked ‘f’ (*p* < 0.05).

Growth of *C. massalongoi* from Glendasan, as measured by a mean increase in colony area (mm^2^), only occurred on the 0 ppm, 3 ppm and to a lesser extent on the 6 ppm treatment plates (Figure 1d). No gemmae were observed on any of the treatment plates. Scheffé post-hoc tests revealed that the 0 ppm colonies were significantly greater in the mean area (mm^2^) than all colonies on the other treatments (*p* < 0.01; Figure 4a). The 3 ppm colonies were not significantly greater in area than any other treatment colonies. Neither the colonies of the 6 ppm treatment plates nor the stems of the 12 ppm, 24 ppm, 48 ppm and 96 ppm treatments registered on the analytical balance and so all these were omitted from the analysis of dry weights. An independent two-sample t-test comparing the mean dry weights of the 0 ppm and 3 ppm treatment replicate colonies was carried out and this revealed a significant difference between the two (*p* < 0.05; Figure 4b).

Growth of *C. massalongoi* from Ross Island only occurred on the 0 ppm and 3 ppm treatment plates (Figure 1e). There was no change in the area of the stems on the other treatments and they were too minuscule to measure on an analytical balance. Gemmae were observed on the 0 ppm and 3 ppm treatment plates only. Scheffé post hoc tests showed that the mean colony area (mm^2^) of the 0 ppm treatment plates was significantly greater than that of all other treatment plates (*p* < 0.001), and that the mean colony area of the 3 ppm plates was significantly greater than all others (apart from 0 ppm) (*p* < 0.001; Figure 4c). An independent two-sample t-test comparing the mean dry weights of the 0 ppm and 3 ppm treatment replicate colonies (Figure 4d) was carried out and this revealed a significant difference between the two (*p* < 0.05).

Growth in the area of *C. massalongoi* from the Gunnislake Clitters population continued steadily on all treatment plates throughout the course of the experiment (Figure 1f). Gemmae were produced on all treatment plates. Scheffé post-hoc tests revealed that the mean colony area on the 0 ppm treatment plates was significantly greater than on the 3 ppm, 12 ppm and 24 ppm plates (*p* < 0.05) and also the 48 ppm and 96 ppm treatment plates (*p* < 0.01). Growth on the 6 ppm treatment plates as measured by mean colony area (mm^2^) was significantly greater than on the 96 ppm treatment (*p* < 0.05) (Figure 4e). However, taking growth as measured by mean dry weight, the 0 ppm treatment was only significantly greater than that of the 96 ppm treatment plates (*p* < 0.05; Figure 4f).

**Figure 5:**
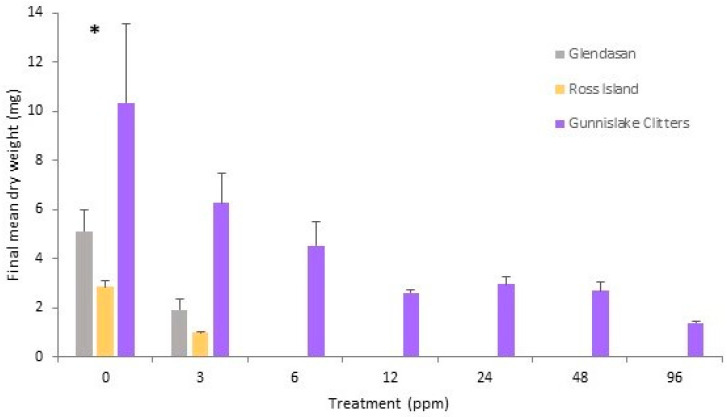
Final mean dry weight (mg) per copper treatment, with standard error bars, at week 20, for *Cephaloziella massalongoi* from all three populations. A significant difference was found among populations within the treatment marked ‘*’ (*p* < 0.05).

Two-way factorial ANOVA analysis showed an overall significant difference among the populations (Table 3). Scheffé post-hoc tests revealed that the final dry weight of the Gunnislake Clitters population was significantly greater than both the Glendasan and Ross Island populations (*p* < 0.01), with no significant difference between the latter two populations. There was also an overall treatment effect and Scheffé post-hoc tests showed that the results of the 0 ppm treatment were significantly greater in dry weight than those of all other treatments (*p* < 0.05). Within the treatments, the final mean dry weight (mg) of the 0 ppm treatment of the Gunnislake Clitters population was significantly greater than that of the Ross Island population and the 3–96 ppm treatments of the other populations (*p* < 0.05). Also, the final dry weight (mg) of the 3 ppm treatment of the Gunnislake Clitters population was significantly greater than that of the Ross Island population (*p* < 0.05) and the 6–96 ppm treatments of the other populations (Figure 5).

### 2.5. Tolerance Indices of C. nicholsonii and C. massalongoi

Tolerance indices (TI) were computed by dividing the dry weight of each replicate of each treatment by the mean dry weight of the 0 ppm treatment per population. Where no dry weight could be measured the tolerance index was given as zero, as no growth occurred in such cases. A Wilcoxon Rank Sum test was carried out to compare the mean tolerance indices per species per treatment; it showed significant differences between *C. nicholsonii* and *C. massalongoi* for the 6 ppm and 12 ppm copper treatments (Table 4).

## 3. Discussion

The main, and unanticipated, outcome of this experiment is that both *Cephaloziella nicholsonii* and *C. massalongoi* grew well, and in some cases significantly better, on the culture plates with zero copper addition. Utmost care was taken when preparing this medium in choosing reagents with no copper contamination, but trace amounts may have been present. However, all plants require copper in small amounts for redox reactions [55]. The fact that these ‘copper mosses’ can survive and thrive on a substrate containing only trace amounts of copper suggests that the reason for their growth and survival at copper mine sites is due to the lack of competition or to other factors. Even if the species do not require elevated copper levels, tolerance to the heavy metal gives them a competitive edge. There is a record of *C. massalongoi* from a wall in Great Britain where there was no apparent copper influence [48] and so it may be possible that the species can survive in areas without copper enrichment. Some of the populations sampled came from former lead and zinc mines which, although they have relatively high amounts of copper, contain higher levels of lead and zinc (Table 1). It has been speculated that ‘copper mosses’ do not possess an elevated nutritional requirement for copper, but are restricted to high copper substrates because they are poor competitors with an unusually high tolerance of copper and/or its associated sulphide-generated acidity [56]. It has also been suggested that metallophyte species are probably able to tolerate very low soil-water pH and a high concentration of sulphate ions in their niches rather than being ‘copper mosses’ [57], and that ‘copper mosses’, including *C. massalongoi,* may be better described as ‘sulphur mosses’ as they occur on substrates rich in copper, iron and zinc sulphides [58]. In this study, some *C. nicholsonii* and *C. massalongoi* populations’ growth showed an incremental decrease across the low-to-high ranging copper treatments. Soil reaction at the field sites in this study varied across a pH range of 5.8–7.03 (Table 1). As well as extreme acidity and high metal and sulphide levels, many other factors could contribute to an area being species-poor and allowing *C. nicholsonii* and *C. massalongoi* to succeed, such as disturbance, minimal soil development and low nutrient levels [2].

Also unanticipated is the variation in survival among the different populations of the same species. *C. nicholsonii* from South Caradon Mine in Cornwall performed better on the 0 ppm treatment plates than on the 24 ppm, 48 ppm and 96 ppm treatment plates, but growth still occurred on the higher copper treatments (less so on the 24 ppm plates however). While *C. nicholsonii* from the Allihies population performed significantly better on the 3 ppm and 6 ppm treatments than on the 48 ppm and 96 ppm in terms of final mean dry weight, growth still occurred on the higher copper treatment. Levels of 6 ppm copper and above have been found to inhibit the growth of *Funaria hygrometrica* and *Marchantia polymorpha* [59] and levels of 20 ppm and above reduced the photosynthetic activity of the copper-tolerant moss *Scopelophila cataractae* [60]. The results of this study suggest that elevated copper is not obligately required by the South Caradon Mine and Allihies populations, but that a high level of tolerance exists in each population.

This is in contrast with the Caim population, where dry weight only increased in the 0 ppm, 3 ppm, 6 ppm and 12 ppm treatment plates. Overall, the Caim population had a final dry weight significantly less than the other two populations across all treatments. The Allihies replicates also had a final dry weight in the 6 ppm treatment significantly higher than that of the Caim replicates. No growth of Caim samples occurred on the higher copper treatment plates of 24 ppm, 48 ppm and 96 ppm copper. The final mean area increase and the final dry weight were greater in the 0 ppm treatment than the other treatments, although not significantly so in the latter measure of growth, which suggests that copper is not required in larger than trace amounts for this population. Some of the plates from this population were infected with fungal contamination and this may have negatively affected overall growth. However, growth did not occur on the higher treatment plates regardless of fungal presence. Caim is a former copper and lead mine. Copper levels in the soil sampled at Caim were relatively high (2513.1 mg kg^−1^), and qualify as being copper contaminated [10], but were lower than the copper level found in the soil samples from Allihies (9877.0 mg kg^−1^), where levels of up to 75,520 mg kg^−1^ have been recorded in solid mine waste [11]. However, lead concentrations in the soil were much greater at Caim (37,006.6 mg kg^−1^), where levels of 5674–85,213 mg kg^−1^ have been previously recorded in solid mine waste [11]. *C. nicholsonii* is also found in Ireland on soils with even less copper enrichment than that at Caim, for example near the former lead mine at Avoca (Tigroney West), Co. Wicklow [25]. It must be borne in mind that the bioavailable content of the total copper content in soils can be relatively low [61]. However, these results suggest that different levels of tolerance to copper have developed in particular populations of *C. nicholsonii*, and that these are dependent—at least in part—on the levels of copper present within the site.

Levels of copper in the soils from the Cornish sites at South Caradon and Gunnislake Clitters were not recorded in this study, but copper levels from Cornish mine spoil soils have been found to be high, for example, 1316–3512 ppm (equivalent to mg kg^−1^) in the acid-extractable fraction [47]. At seven Cornish sites for *C. nicholsonii* and *C. massalongoi*, total copper was found to be in the range of 1030–6930 ppm (using X-ray fluorescence spectrophotometry) with lower levels of lead (377.8–770 ppm) and zinc (30.5–1600 ppm) [3]. A level of up to 36,500 ppm copper has been recorded at a *C. massalongoi* site in Snowdonia [40]. Populations of *C. massalongoi* from sites with the lower amounts of copper in the range over all sites (see Table 1) showed little or no growth on the higher copper treatments. However, colonies derived from the Glendasan population of *C. massalongoi*—which had a lower soil copper content than the soil from Ross Island—were able to grow better on the 3 ppm treatment than colonies derived from Ross Island. The *C. massalongoi* population from Gunnislake Clitters differed from the other two populations in making substantial growth across the whole spectrum of copper treatments; it also showed substantially greater growth on the 0 ppm treatment than the other two populations (significantly more than the Ross Island population (*p* < 0.05)).

According to Lockhart et al. [17], strict metallophytes tend to grow mainly on acidic rather than base-rich substrates. Paton [38] stated that *C. massalongoi* hasn’t been recorded from old copper mines located in limestone areas. However, the population at Ross Island (discovered after Paton [38] was published) is located in an area with limestone bedrock; the soil pH recorded at this site was 6.28 (the range recorded for this species was 5.8–7.03; Table 1). At higher pH values, the level of available copper relative to total copper is lower than that at a lesser pH [27,57,62]. This appears to be evidence that ecotypic variation exists within *C. massalongoi*, as well as *C. nicholsonii*, and that elevated copper levels are not obligatory for the growth of *C. massalongoi*.

No growth of *C. massalongoi* samples taken from Bunmahon or Allihies Mountain Mine occurred on any of the treatments, from 0 ppm to 96 ppm. What sets these two populations apart is puzzling. It may be that the sterilisation procedure damaged them more than the other population samples, but stems remained green for circa 1–2 weeks after sterilisation so this is unlikely. Also, samples from these populations had been collected twice for initiation into the culture, and no growth occurred on either occasion. Interestingly, both sites had the highest levels of copper of all the soil samples collected and analysed (Table 1). Shaw [63] states that the average range of copper in mineral soils from a variety of global locations is 20 ppm, and in organic soils is 350 ppm. The concentration ranges of copper normally encountered in mineral soils is 5–80 ppm and in organic soils (peat) 6–40 ppm according to Allen [9]. The copper levels in the soils from Bunmahon and Allihies greatly exceed these levels and also those of the other copper mine sites in this study. It may be that the *C. massalongoi* populations at Bunmahon and Allihies have adapted to extremely high levels of copper and could not grow even on the highest (96 ppm) level due to insufficient copper. This appears to be further evidence of ecotypic variation within the species.

Other studies on bryophytes—on both facultative and obligate metallophytes—have also yielded results indicating increased tolerances as responses to natural selection and the evolution of adapted ecotypes at elevated levels of metals [26,64,65,66]. However, Shaw et al. [67] found a broad level of apparently inherent tolerance in *Bryum argenteum* in response to changes in lead concentration. Shaw et al. [68] also found little evidence for ecotypic differentiation in *Ceratodon purpureus*: plants from several populations that originated in uncontaminated soils grew as well on mine soils as plants from a mine-site population. Also, no variation was found within five populations of *Scopelophila cataractae* [27], suggesting an innate tolerance that is not lost as one of the populations grew naturally on soils with low levels of copper (albeit with high levels of iron). Shaw [69] found that tolerance of both copper and zinc varied significantly among individuals within a particular population of *Funaria hygrometrica*. The present study consisted of one clone per population per treatment and so did not investigate variation in the ability to tolerate copper among individuals within populations. Further experimental investigation may be merited into tolerance not only of varying copper levels, but also of varying zinc levels, among and between populations.

Tolerance levels tend to be metal-specific [70] and tolerance to one metal does not confer tolerance to another either in angiosperms [71], or in bryophytes [64]. Our field data indicate that *C. massalongoi* and *C. nicholsonii* can simultaneously tolerate abnormally high levels of lead and zinc—as well as copper—in their substratum; again, this merits further investigation.

There is anecdotal evidence that sexual reproduction is inhibited in mosses growing in polluted environments, possibly due to inhibition of gametangia formation or poor development of sporophytes after fertilisation [27,68,70]. However, there is some evidence that increased production of asexual propagules can occur [72]. *C. massalongoi* and *C. nicholsonii* have never been found with sporophytes in Ireland or Britain, although they have been reported for the former species elsewhere ([38], author’s observations). Foliar gemmae are abundantly produced in both of the species. Other European *Cephaloziella* species often produce capsules in abundance, as well as reproducing regularly from foliar gemmae. In this study, no evidence of sexual reproductive structures was observed in culture in either species (unsurprising given the short timeframe of the experiment). Gemmae were produced by *C. nicholsonii* on nearly all plates of the South Caradon Mine population; on the lower copper treatment plates only in the Caim population; and solely on the 0 ppm plates of the Allihies population. Gemmae were produced on all treatment plates of *C. massalongoi* from the Gunnislake Clitters population, and only on the 0 ppm and 3 ppm plates for the Ross Island population; none were produced on any of the plates of the Glendasan population. There appears to be a lot of variation among the populations regarding gemma production, as well as growth, at differing levels of copper.

*C. nicholsonii* samples appeared to have an overall greater tolerance for the 6 ppm and 12 ppm levels of copper than those of *C. massalongoi*. This difference probably reflects the fact that specimens of *C. massalongoi* came from two populations from lead mines, as opposed to only one for *C. nicholsonii*. As the two taxa share so much of their genetic heritage [45,46], there may be similar patterns of ecotypic variation, depending on the metal levels in the substrate. Investigations of tolerances in further populations of both species would determine whether one species is more tolerant than the other to elevated levels of metals. Investigation of sulphide levels would also be worth pursuing.

While experimental investigations under sterile conditions in in vitro cultivation are advantageous in controlling for various factors [73], they cannot replicate in situ growth conditions and processes which may also have an influence on tolerance to heavy metals. Many large populations of both *C. nicholsonii* and *C. massalongoi* are found on slopes or runnels at their sites. These micro-habitats are regularly flushed by surface water and subjected to surface runoff. Consequently, these plants may gain or lose copper and other metal ions from sources other than the immediate substrata, and their external surfaces are regularly washed with water that may or may not have high copper concentrations. These processes deserve further investigation.

Many liverworts have associations with endophytic fungi similar to mycorrhizas in vascular plants [74,75]: the thick-walled rhizoids and the presence of associated fungal endophytes in *Cephaloziella* spp. from metalliferous sites may be a possible factor in their survival there [76]. A study investigating the existence of obligatory gypsophilous bryophytes found that gypsum was not the main factor determining the growth of certain bryophytes in in vitro culture and suggested that other factors in situ may also be involved, such as interactions with other organisms (such as fungi or algae) [77]. These additional factors should be investigated in future research within and among populations.

Another possible explanation for the differences in response to the treatments among the populations of both *C*. *massalongoi* and *C*. *nicholsonii* may lie in taxonomic variation. The genus *Cephaloziella* is taxonomically problematic. This is partly due to the intrinsically diminutive nature of the characters used to distinguish them, and partly because a particular character may not be present in a given colony (e.g., perianths). There is some discrepancy between the characters described by Paton [38] and Schuster [78] and this could be due to genetic differences between populations or even cryptic speciation. DNA barcoding work carried out by researchers in the Royal Botanic Gardens, Edinburgh on liverwort taxa has shown no resolution of the *Cephaloziella* species investigated into acceptable morphological species as defined by the floras, and has concluded that the characters used to distinguish *Cephaloziella* spp. are inadequate; the authors suggest a full taxonomic revision of the genus [79]. Cryptic speciation may be widespread in liverworts [80,81,82] and this could explain differences in ecological preferences among populations, such as those observed in this study. Our findings support the view that a fresh investigation of the genus *Cephaloziella*—combining morphological and molecular techniques—is urgently needed.

As modern mining techniques do not provide suitable habitats for metallophyte bryophytes, their conservation depends on the maintenance and management of the existing old mine sites that contain these rare and ecologically specialised species [14]. Often these sites are considered eye-sores and dangerous to public safety and so are under threat from ‘tidying’, reclamation and coverage with topsoil [16]. However, the presence of these rare species—and many others—means that these sites merit being conserved appropriately for their continued survival [83]. One of the other threats to the specialised niche of *C. nicholsonii* and *C. massalongoi* is neglect, because as the sites are unmanaged they are eventually recolonized by taller, coarser vegetation which reduces and ultimately eliminates the habitat of these specialist metallophytes [14]. Contamination with heavy metals may only be restricted to the surface horizons of the soil near copper mines [84] and copper tends to decrease in the upper soil horizon through leaching over time [85]. One management recommendation is the preservation and promotion of bare, metal-rich substrates that can be utilised by these species. Supplementation of copper to a site where levels have decreased is another management option. Our findings indicate that *C. nicholsonii* and *C. massalongoi* do not have an obligate requirement for raised copper levels, and so copper supplementation may not be appropriate, or at least should be done carefully and only after examination of former levels. Opening up of bare areas (soil scraping) may be sufficient to maintain areas of sparse vegetation so that the species do not become out-competed.

## 4. Materials and Methods

### 4.1. Sampling and Initiation into Culture

Samples of *Cephaloziella nicholsonii* and *C. massalongoi* were collected from each vice-county from which they are known in Ireland and also from three and two sites from Cornwall, Great Britain, respectively. Samples consisted of patches varying in size (~1–3 cm^2^, depending on the size of the colony from which each was taken), and were collected in paper packets stored in plastic bags (to prevent drying) until processed in the laboratory (within 2–7 days). Sampling of the Cornish specimens took place circa one month before the sampling of the Irish populations. D.T.H. verified the identifications and voucher specimens were accessioned to the National Botanic Gardens of Ireland Herbarium (DBN) for each population.

Samples of *C. massalongoi* from two sites—Mountain Mine, Allihies, Co. Cork (sampled from 1 point) and E. of Bunmahon, Co. Waterford (sampled from 3 points)—failed to grow in in vitro cultivation. These sites were resampled c. 18 months later and stems were selected from the samples for initiation into culture. The remainder of the samples from Allihies and Bunmahon were kept in a cool greenhouse at the National Botanic Gardens, Dublin.

Cultures of *C. nicholsonii* from Allihies, Caim, Co. Wexford and South Caradon Mine, Cornwall and cultures of *C*. *massalongoi* from Glendasan, Co. Wicklow, Ross Island, Co. Kerry and Gunnislake Clitters, Cornwall were chosen for the main experimental investigation because they represented two Irish and one British population for each species. Two additional chosen populations for *C. massalongoi*, from the Bunmahon and Allihies sites, were those that had failed to grow in culture previously. Details of the habitat and mining history of the sites are presented in Table 1.

In the laboratory, stems of *C. nicholsonii* and *C. massalongoi* were extracted from each sampled patch with a fine forceps. Under sterile conditions in a laminar flow cabinet, the specimens were each placed on a 55 mm filter paper positioned within a Sartorius stedim 16,309 vacuum flask and filter system (previously autoclaved at 120 °C and 15 psi), connected to a vacuum pump. Specimens were washed through with sterile deionised water five times and given two washes with a sterilising agent solution; the second wash was allowed to stand to immerse the specimen for three minutes and then washed through five times with sterile deionised water. The sterilising agent used was a 1:10 dilution of stock of 7% calcium hypochlorite (Ca(ClO)_2_). Tween, a polysorbate surfactant, was added to dilution solutions as a detergent and wetting agent to improve contact between the tissue and the disinfectant. Sterilised stems were then placed carefully onto petri dishes containing modified Parker medium [86]. The modified amounts (mg) of macro- and microelements contained per litre of this medium are outlined in Table 5. The medium was made to 1 litre, solidified with 10 g agar, and the pH was adjusted to 5.8 before autoclaving at 120 °C and 15 psi for 20 min to sterilise.

This medium contains 0.4 ppm copper as copper sulphate pentahydrate (CuSO_4_·5H_2_O) and 2 ppm zinc as zinc sulphate heptahydrate (ZnSO_4_·7H_2_O). All populations were grown on this medium for a number of months, to eliminate any pre-treatment effects or possible environmental effects, in a micropropagation growth room at 22 ± 2 °C and 45–50% relative humidity, with an irradiance of 58.6 watts per second on a 16/8 h light/dark cycle.

### 4.2. Growth Media for Experimental Investigation

The base growth medium for the experimental investigation was prepared as above, but without any copper-containing ingredient. The reagents used to make up the base medium were of high analytical grade and chosen for the lack of copper contamination within them in order to minimize the amount of copper in the base medium. For the copper treatments, the base medium (0 ppm/trace Cu) was supplemented with 3 ppm, 6 ppm, 12 ppm, 24 ppm, 48 ppm and 96 ppm copper as CuSO_4_·5H_2_O (copper sulphate pentahydrate). All treatment media were prepared on the same day and autoclaved together at 15 psi for 20 min before they were poured into labelled sterile plastic Petri dishes.

### 4.3. Experimental Procedure

Stems of both species were extracted from clonal patches on existing plates using a fine forceps in a laminar flow cabinet. Stems taken ranged in length from 1.5–4.0 mm. Each stem was placed, with a sterile needle, on the center of a petri dish containing the 0 ppm, 3 ppm, 6 ppm, 12 ppm, 24 ppm, 48 ppm or 96 ppm treatment media with four replicate plates per treatment. An attempt had been made to weigh some individual stems on an analytical balance (OHAUS GA110; with four decimal place accuracy) at the commencement of the experiment, but the stems were too minuscule to register.

Stems of *C. massalongoi* from Bunmahon and Allihies that had been growing in the cool greenhouse from sampled patches were extracted using a fine forceps and sterilised using the procedure described above, but using 0.5% sodium dichloroisocyanurate (NaDCC) as the sterilizing agent, not 0.7% Ca(ClO)_2_. Stems were then placed, with a sterile needle, on the center of the treatment plates with three replicates per treatment. All experimental cultures were maintained in the growing room at the conditions described above. They were arranged in three replicate blocks which were repositioned every two weeks to avoid any positional effects.

Eight days after commencement, all plates were inspected for infection with an Olympus^®^ SZ61 stereo microscope (Olympus Corporation, Tokyo, Japan) with a uEye ^®^ camera (IDS Imaging Development Systems Incorporated, Hessen, Germany) attached and photographed using QuickPhoto Micro version 2.3 software (Promicra) attached to the microscope. All plates were inspected and photographed a further 16 days later and then every 14 days thereafter until completion of the experiment, a total of 20 weeks after commencement. The area of each colony was photographed from above each time. At the conclusion of the experiment, further photographs were taken on an Olympus^®^ BX51 compound microscope attached to the uEye ^®^ camera. All areas were measured using ImageJ software version 1.46r [87].

At the experiment’s conclusion at 20 weeks, the colonies were weighed for the fresh weight (mg) on an electronic analytical balance. The colonies lifted off the agar medium easily in most cases. Any medium stuck to the bottom was carefully scraped off using a scalpel. Not all gemmae remaining on the agar could be collected, however. Each colony was then placed in a labelled brown paper packet and these were put in a drying oven at 40 °C for 48 h. After such time the colonies were re-weighed on the analytical balance in order to obtain final dry weight (mg) measurements.

### 4.4. Soil Sampling and Analysis

One soil sample was collected from immediately below where each liverwort sample was taken in the field and frozen until ready for analysis. Each sample was sieved through a 2 mm copper-free sieve and dried at 40 °C for 48 h in a drying oven. Dried samples were sent to Euro Environmental Services (now Fitz Scientific) Laboratories, Co. Louth, Ireland for total copper, lead and zinc analysis. At this laboratory, samples were extracted using aqua regia (nitro-hydrochloric acid) in a midblock digester. Aqua regia was added to 1 g of soil per sample and then digested. After digestion the samples were filtered, diluted to 100 mL and analysed by inductively coupled plasma atomic emission spectroscopy.

Analysis for pH was carried out by the first author. Two replicates of 10 g of dried and sieved soil were measured on an analytical balance (OHAUS, Adventurer SL) and placed in a 100 mL glass beaker. 20 mL of deionised water was added to each to obtain a soil:water ratio of 1:2 [88]. The ‘slurry’ solutions were stirred with a glass rod and left to settle for 1–2 h. The samples were then analysed using a pH meter (Jenway 3310) that was calibrated with pH 4 and 7 buffers before the first sample and was re-calibrated after every 10 samples.

### 4.5. Data Handling and Analysis

Statistical analysis was carried out on the response variables of growth measured as final colony area (mm^2^) and growth measured as final dry weight (mg) at the conclusion of the experiment. Data were tested for normality using the Shapiro-Wilks test [89] on R software (version 2.15.1). Differences between the final mean colony area (mm^2^) and final mean dry weight (mg) per treatment per population were analysed by one-way ANOVA and a Scheffé post-hoc test using DataDesk (version 6.0) with the null hypothesis that there was no difference in growth between treatments for a given population. Comparisons among populations of each species subjected to the different copper treatments were analysed by two-way factorial ANOVA and a Scheffé post-hoc test for the significance of interactions using DataDesk. To compare the growth responses of the two species to each other tolerance indices were calculated by dividing the growth on each copper addition treatment replicate by the mean growth on the 0 ppm plates. The differences between the species were then analysed by Wilcoxon Rank Sum tests for each treatment level using R software.

## 5. Conclusions

This in vitro experiment resulted in the unanticipated outcome that the metallophyte liverworts *Cephaloziella nicholsonii* and *C. massalongoi* can survive without copper supplementation, and in some cases performed significantly better on treatments with zero (trace) copper present. The variation of tolerance levels among the populations to the copper treatment levels (0 ppm, 3 ppm, 6 ppm, 12 ppm, 24 ppm, 48 ppm and 96 ppm) was another interesting finding, with populations at locations with higher in situ copper soil levels being able to tolerate higher copper levels in ex-situ conditions. Variation in gemma production was also observed. These results suggest that copper supplementation may not be appropriate as an in situ conservation measure. The results suggest that ecotypic variation occurs within both species. However, other factors such as in situ levels of other heavy metals and sulphides, micro-habitat conditions and interaction with fungal endophytes could also be relevant and merit further investigation. Taxonomic variation is a further possible explanation for the interpopulation differences in treatment response. This study highlights the need for an in-depth taxonomic revision of the *Cephaloziella* genus.

## Figures and Tables

**Table 1:** Details of former mine sites of *Cephaloziella nicholsonii* and *C. massalongoi* populations from which soil samples were taken (grid reference (ref.), bedrock type, metal(s) mined, i.e., copper (Cu), lead (Pb), tin (Sn), arsenic (Ar), tungsten (W); years (yrs) worked) and soil analyses for pH (units), copper, lead and zinc (mg kg^−1^). ND = no data.

Species	Population	Grid Ref.	Bedrock	Metal(s) Mined	Yrs Worked	pH	Cu	Pb	Zn
*Cephaloziella nicholsonii*	Caim, Co. Waterford	S885408	Metamorphic	Cu & Pb	1815–1855	5.83	2513	37,004	1495
	Mountain Mine, Allihies, Co. Cork	V589456	Sandstone	Cu	1813–1918	5.81	9877	129	162
	South Caradon Mine, Cornwall	SX264698	Granite	Cu	1837–1885	ND	ND	ND	ND
*Cephaloziella massalongoi*	Ross Island, Co. Kerry	V945880	Limestone	Cu & Pb	1804–1810	6.28	3815	9911	12,647
	Glendasan, Co. Wicklow	T098981	Granite, Schist	Pb	1807–1900	7.03	478	30,522	14,502
	Gunnislake Clitters, Cornwall	SX422720	Granite	Cu, Sn, Ar & W	1820–1919	ND	ND	ND	ND
	Bunmahon, Co. Waterford	X443986	Slate	Cu	1730–1907	5.80	12,528	34	81
	Mountain Mine, Allihies, Co. Cork	V590458	Sandstone	Cu	1813–1918	5.99	6019	52	32

**Table 2:** Two-way factorial ANOVA table for the final mean dry weight (mg) of replicates per copper treatment of *Cephaloziella nicholsonii* populations. Dry weights (mg) of the 24 ppm, 48 ppm and 96 ppm treatments that were too small to register on the analytical balance were entered as 0.00001 for the purposes of this analysis.

Source	Degrees of Freedom	Sum of Squares	Mean Square	F-Ratio	*p*
Constant	1	162.563	162.563	239.34	≤0.0001
Population	2	23.2821	11.641	17.139	≤0.0001
Treatment	6	78.5549	13.0925	19.276	≤0.0001
Population × Treatment	12	26.9134	2.24278	3.3021	0.0020
Error	42	28.5267	0.679206		
Total	62	157.277			

**Table 3:** Two-way factorial ANOVA table for the mean dry weight (mg) of replicates per copper treatment of *Cephaloziella massalongoi* populations. Dry weights (mg) of the 24 ppm, 48 ppm and 96 ppm treatments that were too small to register on the analytical balance were entered as 0.0001 for the purposes of this analysis.

Source	Degrees of Freedom	Sum of Squares	Mean Square	F-Ratio	*p*
Constant	1	244.864	244.864	122.65	≤0.0001
Population	2	215.456	35.9094	17.986	≤0.0001
Treatment	6	188.919	94.4595	47.312	≤0.0001
Population × Treatment	12	41.3471	3.44559	1.7258	0.0954
Error	42	83.8533	1.99651		
Total	62	529.576			

**Table 4:** Mean tolerance indices per copper treatment per species (*Cephaloziella nicholsonii* and *C*. *massalongoi*) with *p*-value results of Wilcoxon Rank Sum tests: (ns) = non-significant; ** = significant at *p* < 0.01.

Treatment	*Cephaloziella* *nicholsonii*	*Cephaloziella* *massalongoi*	*p*
3 ppm	0.79 (0.19)	0.44 (0.06)	0.26 (ns)
6 ppm	0.81 (0.23)	0.15 (0.08)	0.007 **
12 ppm	0.51 (0.11)	0.08 (0.04)	0.005 **
24 ppm	0.22 (0.07)	0.10 (0.05)	0.09 (ns)
48 ppm	0.03 (0.01)	0.09 (0.04)	0.67 (ns)
96 ppm	0.14 (0.04)	0.05 (0.02)	0.09 (ns)

**Table 5:** Amounts (mg litre^−1^) of macroelements and microelements in culture medium.

	Chemical Name	Chemical Formula	mg Litre^−1^
Macroelements	Ammonium nitrate ^1^	NH_4_NO_3_	125.0
	Potassium dihydrogen phosphate ^1^	KH_2_PO_4_	500.0
	Magnesium sulphate ^1^	MgSO_4_	120.0
	Calcium chloride ^1^	CaCl_2_	20.0
Microelements	Sequestrene 330 ^1^	C_14_H_18_FeN_3_Na_2_O_10_·× H_2_O	14.0
	Boric Acid ^1^	H_3_BO_3_	11.4
	Zinc sulphate ^2^	ZnSO_4_·7H_2_O	8.82
	Copper sulphate ^2^	CuSO_4_·5H_2_O	1.57
	Manganese sulphate ^2^	MnSO_4_·4H_2_O	0.0223
	Sodium molybdate ^2^	Na_2_MoO_4_·2H_2_O	0.0250
	Cobalt chloride ^2^	CoCl_2_·6H_2_O	0.0025

^1^ Stock solution was prepared by adding the mg litre^−1^ × 10 amounts to 1 litre of deionised water. The stock was then divided into ten 100 mL volume bags and stored at −20 °C and defrosted for use, i.e., 100 mL per litre final medium. ^2^ Stock solution was prepared by adding the mg litre^−1^ × 1000 amounts to 1 litre of acidified water (999 mL deionised H_2_O and 1 mL sulphuric acid (H_2_SO_4_)). Stock solution was stored in plastic bags at −20 °C and defrosted for use of 1 mL per litre final medium.

## Data Availability

The datasets used and/or analyzed during the current study are available from the corresponding author on request.
